# Epidemiology of hepatitis A virus in Africa among persons aged 1–10 years: a systematic review protocol

**DOI:** 10.1186/s13643-015-0112-5

**Published:** 2015-09-26

**Authors:** Tiwonge J. Kanyenda, Leila H. Abdullahi, Gregory D. Hussey, Benjamin M. Kagina

**Affiliations:** Vaccines for Africa Initiative, Division of Medical Microbiology and Institute of Infectious Disease and Molecular Medicine, University of Cape Town, Cape Town, South Africa

## Abstract

**Background:**

Africa is considered an area of high endemicity for hepatitis A virus infection. However, in the past two decades, tremendous progress has been made in improving water sources and sanitation which are risk factors for hepatitis A virus infection. Recent studies suggest that several African countries could be in epidemiological transitions due to the evident socio-economic development. As a result, there may be a decrease in the exposure to and infection with hepatitis A virus at an early age. Understanding and mapping the shifting epidemiology is vital in developing control measures against the disease. We are conducting a comprehensive systematic review study to document the current burden of hepatitis A virus infection in Africa.

**Methods:**

Our population of interest is children between 1 and 10 years in any African country. We will select cross-sectional, case-control, and cohort studies that have tested hepatitis A virus infection by serological confirmation of antibodies against the virus. We will search for eligible studies published without language restrictions from PubMed, Scopus, Africa-wide, Web of Science, and WHOLIS as well as the reference lists of the relevant articles. Two authors will independently review the search outputs, select eligible articles, and extract pre-defined study outcomes. Inconsistencies will be resolved by discussion and consensus among the authors.

Data will be extracted using a standardised data collection form. Trends in the prevalence and/or incidence will be evaluated by urban and rural setting if sufficient data is available. Where there is sufficient homogeneity between studies, meta-analysis will also be conducted, otherwise the results will be presented in a narrative format.

**Discussion:**

The systematic review will generate up-to-date information on the current burden of hepatitis A virus in Africa. This information may have implications on policy for hepatitis A vaccination on individual African countries.

**Systematic review registration:**

CRD42015023764

## Background

Hepatitis A is a common viral infection that is associated with poor access to the following: safe and clean drinking water, sanitation, and hygiene [1–3]. Globally, 1.4 million cases of hepatitis A virus (HAV) occur annually with a majority of the cases concentrated in the less developed countries where the risk factors facilitate transmission [3]. In the past two decades, the HAV cases have declined dramatically in many parts of the world due to improved socio-economic developments and partly due to the availability of vaccines against the virus [2, 4, 5]. The advances in hygiene and sanitation have resulted to an increased age at which children get first exposure to or infection with HAV. In order to develop and improve the strategies for HAV infection control, it is important to constantly assess the epidemiology of the disease. Hence, we are conducting this review to evaluate the burden of HAV infection in Africa.

A systematic review on HAV infection burden in the world by the World Health Organisation (WHO), conducted in 2009, showed that infection rates remain high in most African countries [3]. Africa is thus still considered a continent of high HAV endemicity, with most of the population infected during early childhood, an age at which the infection is characterised by asymptomatic illness [3, 4]. Infection with HAV during early childhood results to a majority of the population acquiring lifelong immunity to the disease. It is not necessary to have routine immunisation programmes against HAV in high-endemic settings. In contrast, low and intermediate HAV endemic countries have a majority of children who do not get exposed during early childhood which can result into a large population of susceptible adolescents and adults later in life. In these settings, routine immunisation against HAV is recommended [3].

Given that the hepatitis A disease severity is associated with age, there is a risk of outbreaks and other economic implications on the community and the health system if HAV infection occurs later, after the childhood period [3, 6].

The 2009 systematic review by WHO showed the infection rates for HAV to be high in Africa [3]. However, recent studies suggest a general decline in HAV infection in some countries [4, 7]. In Africa, urban areas are transitioning to low rates of HAV infection while high rates of infection are still prevalent in rural areas, particularly among low social economic classes [2, 6, 8. A 2008 study in Egypt confirmed the HAV infection decline in children from low social class having 81 % prevalence compared to those of high class with a prevalence rate of 27.3 % [1]. This change in HAV endemicity could be attributed to socio-economic development that has occurred in the last decade [3]. Understanding and mapping the shifting disease epidemiology from childhood to adulthood is important for the development and review of hepatitis A vaccine policies in Africa.

In Latin America, evidence of epidemiological transition of HAV infection has been used to influence vaccine policy change [9, 10]. Argentina, as an example, introduced a single-dose universal vaccination against HAV infection in 2005 in response to evidence of shifting epidemiology from high endemic to medium endemic [11]. Brazil and Chile who have confirmed similar epidemiological trends to Argentina are evaluating the possibility of introducing targeted vaccination programs [5, 12]. These examples show the need to understand the current HAV epidemiology in order to design national specific control strategies.

### Rationale

The possible shift in HAV epidemiology characterised by most infection occurring late in childhood and adulthood is not well understood in Africa. The change in HAV endemicity in African countries will have vaccine policy implications towards the control of HAV. Currently, most Expanded Programme on Immunisation (EPI) on the continent do not include the hepatitis A vaccine in the routine schedule, under the presumption that immunity is acquired early in life [3, 4]. While infection in early life is asymptomatic, the disease severity increases with age. As such, an investigation of the possible shift in HAV epidemiology, particularly in childhood is important to inform policy on HAV control strategies.

An up-to-date profile on the changing epidemiology of the disease will provide empirical evidence that can guide policy makers when developing evidence-based hepatitis A vaccination and prevention strategies in Africa. Although the systematic review by WHO covered all countries of the world, studies were only sourced from a single database (PubMed) and only 23 countries from Africa were included at the time. We therefore propose an up-to-date and more comprehensive systematic review that will identify all relevant studies published on the topic in several databases. Our systematic review builds on to the review by WHO in 2009 with a focus on African continent. This study will seek to determine the current age-related HAV seroprevalence, monitor the trends in the disease burden, and document any emerging epidemiological changes that have occurred in the past 10 years. The past 10-year period is characterised by significant continental improvements in sanitation and access to clean water.

### Objectives

1. Estimate the endemicity of HAV infection among 1–10 years old in different countries in Africa
2. Estimate the age-specific HAV prevalence and/or incidence in different African countries
3. Evaluate the trend of HAV prevalence and/or incidence in different African countries in the last decade

## Methods

### Types of studies

We will select all studies that have a clearly defined prevalence and/or incidence rate which is defined as presence of anti-HAV antibodies by confirmed blood tests within a clearly defined population. These include the following:

1. Cross-sectional studies
2. Clinical studies
3. Case-control studies
4. Longitudinal studies

### Types of participants

The review will include studies involving all children older than a year and up to 10 years in any African country. Children under the age of 1 year normally acquire passive immunity (antibodies) from their mothers that wanes after 6 months. Therefore, studies with participants under the age of 1 year will be excluded.

The 10-year cutoff point will be used as this is the period when substantial improvements in hygiene, water, and sanitation which are risk factors for HAV infection have occurred following the adoption of the Millennium Development Goals (MDGs) and the establishment of global initiatives targeting water and sanitation improvements^1^ [13, 14]. As such, studies involving participants older than 10 years will be excluded.

### Inclusion and exclusion criteria for participants

#### Inclusion criteria

- Persons aged 1 up to 10 years
- All studies with clear serological confirmation of anti-HAV antibodies by blood tests
- Studies with defined denominator
- Studies must have reported seroprevalence for at least two different age range within 1–10 years of age.

#### Exclusion criteria

- Published after May 2015 or before 2005
- Not conducted on African continent
- No defined serological confirmation
- Case reports
- Studies focusing on high-risk groups, people with human immunodeficiency virus (HIV) or liver disease

### Types of interventions

Not applicable.

### Types of outcome measures

#### Primary outcomes

1. Prevalence of HAV.

Prevalence will be defined as presence of anti-HAV (IgG) antibodies in serologic blood samples taken from population-based samples.

#### Secondary Outcomes

1. Age-specific prevalence of HAV
2. Trends in hepatitis A virus prevalence in Africa.
3. Incidence rate of HAV in Africa

### Search methods for identification of studies

We will select studies conducted in any African country where population studies of anti-HAV antibodies were conducted without language restrictions and with time limits of between 2005 and 2015. The search strategy will use both medical subject heading (MeSH) terms and text words which will be adapted for each database.

Eligible studies will be searched in PubMed, Scopus, Africa-Wide, Web of Science, and WHOLIS. We will also search for other relevant articles from the reference lists of publications. Appendix 1 shows our proposed search strategy, applied in PubMed database to identify relevant studies.

### Data collection and analysis

#### Selection of studies

The primary author will screen the search output by first reading the titles and abstracts, guided by the inclusion and exclusion criteria described above. Then, the first and second authors will independently screen the full articles of potentially eligible articles to evaluate whether they meet the inclusion criteria of population, condition, study setting, and outcomes. Inconsistencies in the list of eligible studies will be resolved through discussion and consensus and if need be, the opinion of the other last two authors will resolve any disagreements.

#### Data extraction and management

Data will be extracted from the text, tables, and figures onto a standardised data extraction form *(*Appendix 2*)*. In cases of unclear data or uncertainty in eligibility, we will contact the corresponding authors of the studies selected to get clarification of the unclear information. The following data points will be extracted from the studies that meet our eligibility criteria.

- Study characteristics: period, design, objectives, and inclusion criteria
- Study population: country, setting, and denominators
- Diagnostic methods: confirmed blood test for anti-HAV antibodies
- Prevalence or seroprevalence of HAV: confirmed cases
- Incidence rate of HAV
- Patient characteristics: age of the children (mean or median age as presented in the included studies)

#### Assessment of risk of bias

The quality of studies included in the review will be assessed using a scoring scale tool that was adapted from the Hoy et al. guidelines for evaluating prevalence studies (Table 1) [15]. A quality sum score will be calculated from the external and internal validity items in order to determine eligibility of articles and to assess consistency between the study authors [16].

**Table 1:** Quality assessment criteria

Items	Quality score
External validity	
1. Was the study’s target population a close representation of the national population in relation to relevant variables?	(1 point)
2. Was the sampling frame a true or close representation of the target population?	(1 point)
3. Was some form of random selection used to select the sample OR was a census undertaken?	(1 point)
4. Was the likelihood of non-response bias minimal?	(1 point)
Total	(4 points)
Internal validity	
1. Were data collected directly from the participants (as opposed to a proxy)?	(1 point)
2. Was an acceptable case definition used in the study?	(1 point)
3. Was the study instrument that measured the parameter of interest shown to have validity and reliability?	(1 point)
4. Was the same mode of data collection used for all participants?	(1 point)
5. Was the length of the shortest prevalence period for the parameter of interest appropriate?	(1 point)
6. Were the numerator(s) and denominator(s) for the parameter of interest appropriate?	(1 point)
Total	(6 points)

#### Dealing with missing data

For any included studies with missing data, we will contact the corresponding author of the study to provide us with the missing data points. In situations where we do not get the missing data, we will describe the missing data and examine the extent to which the missing data may impact the results.

#### Assessment of heterogeneity

The included studies will be assessed for heterogeneity using the chi-squared test of homogeneity (significant for *P* < 0.1) and quantified using the *I*-squared statistic [17]. In instances where there is extensive heterogeneity between the studies such that statistical pooling is not feasible, the results will be presented in a narrative format. Where homogeneity exists between studies (*I*-squared statistic <50 %), a Mantel-Haenszel random effects model will be used to pool data in a meta-analysis. We will use meta-regression to determine factors systematically associated with HAV infection. We will include income status of the countries as classified by World Bank, urban versus rural settings, and diagnosis criteria in our meta-regression models.

#### Assessment of reporting biases

Publication bias will be assessed using funnel plots.

#### Data synthesis

The results from the studies will be reported as prevalence and/or incidences summarised by country and the year the study was conducted. To take into account the between-study variability, the data will be combined by random effects analysis using the restricted maximum likelihood estimation method. In addition, a meta-regression will be done to explore the association between HAV infection prevalence and time. A trend analysis using the year the study was conducted will be done to investigate the time trends and the infection rates in Africa. STATA software (STATA Corporation, College Station, TX, USA) will be used to perform the statistical calculations on the prevalence and/or incidences data.

#### Subgroup analysis

Subgroup analyses will be conducted if possible on the following: income status of the countries as classified by World Bank [18, 19], urban versus rural settings, and diagnosis criteria. In addition, subgroup analysis will be performed based on the WHO classification (WHO AFRO versus WHO EMRO) as these regions have different disease epidemiology profiles. We may conduct additional analysis based on region, for instance West Africa, Southern Africa, and East Africa, if we have sufficient data.

#### Sensitivity analysis

The effect of the sample size will be assessed through multiple sensitivity analyses which will evaluate the effect of excluding some studies that did not meet the study criteria. In addition, we will also conduct a sensitivity analysis to establish if the results are influenced by methodological differences.

#### Reporting of the review

The findings in our systematic review will be summarised in a flow diagram that will outline the selection process as per PRISMA guidelines for reporting systematic reviews. This will also include the list of excluded studies and the reasons for exclusion. In-text descriptions will be used to describe the qualitative data in the studies.

## Discussion

Although it is presumed that the HAV infection is highly prevalent during early childhood in Africa, the current epidemiological changes present new challenges for control of the disease, especially in adults who may have severe episodes [3, 4]. The growing population of susceptible adults that evade infection in childhood will result to an increase in the occurrence of clinical disease and potential epidemics. It is well established that infection in adulthood increases the risk of morbidity and mortality from the disease [3, 6]. With the current strategy of no vaccination, management of clinical illness will create a significant burden on the fragile health systems which are coping with HIV/AIDS, tuberculosis, malaria, and other infectious diseases. In the long term, this is not a sustainable approach as the economic cost of managing HAV infection will be limited due to resource constraints in the health sector. The burden of the disease on individuals and households will also increase, as infection can lead to losses in productivity [3, 20]. The economic and individual burden of HAV infection can be mitigated by vaccination programs that correspond to the epidemiological pattern of the disease.

Given that the prevalence of HAV is closely associated with the level of development, we would expect further decline in HAV infection in most African countries as they continue implementing strategies that lead to improvements in water, hygiene, and sanitation as part of the United Nations post-2015 Sustainable Development Goals (SDGs) [21]. With the current strategy of no vaccination, Africa sits on an impending HAV epidemic if epidemiology of the disease is not well understood and appropriate measures for control adopted. The lack of systematic evidence showing the changing trends limits the adjustment of current policy on control and development of future policy on prevention. Therefore, this systematic review is important to highlight the epidemiological trends of HAV infection which would inform decision makers on HAV infection control and prevention in African countries.

## Appendix 1

**Table 2:** Search strategy

Query number	Search term
#1	hepatitis a[MeSH Terms] OR “hepatitis a”[All Fields]
#2	(((((((“hepatitis a virus”[MeSH Terms] OR “hepatitis a virus”[All Fields]) OR (“hepatitis a”[MeSH Terms] OR “hepatitis a”[All Fields] OR (“infectious”[All Fields] AND “hepatitis”[All Fields]) OR “infectious hepatitis”[All Fields])) OR ((“virology”[MeSH Terms] OR “virology”[All Fields] OR “viral”[All Fields]) AND (“hepatitis”[MeSH Terms] OR “hepatitis”[All Fields] OR “hepatitis a”[MeSH Terms] OR “hepatitis a”[All Fields]))) OR (“hepatitis a virus”[MeSH Terms] OR “hepatitis a virus”[All Fields] OR “hav”[All Fields])) NOT (“hepatitis b”[MeSH Terms] OR “hepatitis b”[All Fields])) NOT (“hepatitis c”[MeSH Terms] OR “hepatitis c”[All Fields] OR “hepacivirus”[MeSH Terms] OR “hepacivirus”[All Fields])) NOT (“hepatitis d”[MeSH Terms] OR “hepatitis d”[All Fields])) NOT (“hepatitis e”[MeSH Terms] OR “hepatitis e”[All Fields])
#3	#1 OR #2
#4	((((“seroepidemiologic studies”[MeSH Terms] OR (“seroepidemiologic”[All Fields] AND “studies”[All Fields]) OR “seroepidemiologic studies”[All Fields] OR “seroprevalence”[All Fields]) OR (“epidemiology”[Subheading] OR “epidemiology”[All Fields] OR “epidemiology”[MeSH Terms])) OR (“epidemiology”[Subheading] OR “epidemiology”[All Fields] OR “prevalence”[All Fields] OR “prevalence”[MeSH Terms])) OR (“epidemiology”[Subheading] OR “epidemiology”[All Fields] OR “incidence”[All Fields] OR “incidence”[MeSH Terms])) OR burden[All Fields]
#5	#3 AND #4
#6	(africa[tw] OR africa’[tw] OR africa’s[tw] OR africa1[tw] OR africa2[tw] OR africaans[tw] OR africacollaborations[tw] OR africae[tw] OR africaeaustralis[tw] OR africahiv[tw] OR africaid[tw] OR africaid’s[tw] OR africain[tw] OR africaine[tw] OR africaine’s[tw] OR africaines[tw] OR africains[tw] OR africal[tw] OR africam[tw] OR africamum[tw] OR african[tw] OR african’[tw] OR african”[tw] OR african’s[tw] OR african1[tw] OR african2[tw] OR africana[tw] OR africanae[tw] OR africanalleles[tw] OR africanamerican[tw] OR africanan[tw] OR africanane[tw] OR africananes[tw] OR africanasian[tw] OR africanastrongylus[tw] OR africancalotropis[tw] OR africander[tw] OR africanders[tw] OR africane[tw] OR africanendemic[tw] OR africanene[tw] OR africanenes[tw] OR africanensis[tw] OR africanenvironment[tw] OR africaner[tw] OR africanes[tw] OR africani[tw] OR africanised[tw] OR africanism[tw] OR africanist[tw] OR africanists[tw] OR africanity[tw] OR africanium[tw] OR africanizada[tw] OR africanization[tw] OR africanization’[tw] OR africanize[tw] OR africanized[tw] OR africanized’[tw] OR africanizing[tw] OR africanjournal[tw] OR africannum[tw] OR africano[tw] OR africanoides[tw] OR africanol[tw] OR africanos[tw] OR africanoside[tw] OR africanpatients[tw] OR africanpiper[tw] OR africans[tw] OR africans’[tw] OR africanton[tw] OR africantrinervitermes[tw] OR africantriol[tw] OR africanum[tw] OR africanum’[tw] OR africanumsp[tw] OR africanumt[tw] OR africanus[tw] OR africanus’[tw] OR africanusgen[tw] OR africanz[tw] OR africare[tw] OR africarice[tw] OR africas[tw] OR africasia[tw] OR africative[tw]) OR Algeria[tw] OR Angola[tw] OR Benin[tw] OR Botswana[tw] OR Burundi[tw] OR Cameroon[tw] OR Chad[tw] OR Comoros[tw] OR Congo[tw] OR Djibouti[tw] OR Egypt[tw] OR Eritrea[tw] OR Ethiopia[tw] OR Gabon[tw] OR Gambia[tw] OR Ghana[tw] OR Guinea[tw] OR Jamahiriya[tw] OR Jamahiriya[tw] OR Kenya[tw] OR Lesotho[tw] OR Liberia[tw] OR Libya[tw] OR Libya[tw] OR Madagascar[tw] OR Malawi[tw] OR Mali[tw] OR Mauritania[tw] OR Mauritius[tw] OR Mayotte[tw] OR Morocco[tw] OR Mozambique[tw] OR Mozambique[tw] OR Namibia[tw] OR Niger[tw] OR Nigeria[tw] OR Principe[tw] OR Reunion[tw] OR Rwanda[tw] OR Senegal[tw] OR Seychelles[tw] OR Somalia[tw] OR Sudan[tw] OR Swaziland[tw] OR Tanzania[tw] OR Togo[tw] OR Tunisia[tw] OR Uganda[tw] OR Zaire[tw] OR Zambia[tw] OR Zimbabwe[tw]
#7	#5 AND #6
#8	2005/01/01[PDAT] : “2015/05/31”[PDAT]
#9	#7 AND #8
#10	AND “humans”[MeSH Terms]
#11	#9 AND #10

## Appendix 2

**Table 3:** Data collection form

Review title	Epidemiology of hepatitis A virus in Africa among persons aged 1–10 years: a systematic review protocol		
Study ID	Surname of first author and year article was published e.g., John 2010		
1. General information			
Date form completed (dd/mm/yyyy)			
Name of person extracting data			
Full reference of article			
Study author contact details			
Publication type (e.g., report, abstract, full article)			
Study funding sources			
Conflict of interest			
Notes:			
2. Study eligibility			
Study characteristics	Eligibility criteria	Yes/no	Location in text
Period	Between 2005 and May 2015		
Setting	African population		
Participants	Above 1 up to 10 years		
Condition	Positive anti-HAV antibodies		
Type of outcome measure	Prevalence and/or incidence not case reports		
Eligibility decision	Include		
	Exclude		
Reason for exclusion			
Notes:			
Do not proceed if study excluded from review			
3. Participants			
	Description		Location in text
Country			
Study setting e.g., urban, rural, hospital based			
Inclusion criteria (in the study)			
Exclusion criteria (in the study)			
Informed consent			
Total population at start of study			
Age of study population			
Sex			
Other relevant socio-demographics			
Notes:			
4. Methods			
	Description		Location in text
Aim of study			
Study design			
Unit of allocation (individuals, cluster, groups)			
Start date			
End date			
Total study duration			
Type of diagnostic test			
Ethical approval obtained for study			
Notes:			
5. Risk of bias assessment			
Items	Quality score		Total score
External validity			
1. Was the study’s target population a close representation of the national population in relation to relevant variables			(1 point)
2. Was the sampling frame a true or close representation of the target population?			(1 point)
3. Was some form of random selection used to select the sample, OR was a census undertaken?			(1 point)
4. Was the likelihood of non-response bias minimal?			(1 point)
Total (4 points)			
Internal validity			
1. Were data collected directly from the participants (as opposed to a proxy)?			(1 point)
2. Was an acceptable case definition used in the study?			(1 point)
3. Was the study instrument that measured the parameter of interest shown to have validity and reliability?			(1 point)
4. Was the same mode of data collection used for all participants?			(1 point)
5. Was the length of the shortest prevalence period for the parameter of interest appropriate?			(1 point)
6. Were the numerator(s) and denominator(s) for the parameter of interest appropriate?			(1 point)
Total			(6 points)
Notes:			
6. Outcomes			
Outcomes	Description as in article		Location in text
Case definition			
Unit of measurement			
Number of cases (prevalence)			
Total number of cases/total pop	# of cases	Total pop	
Number of new cases (incidence)			
Total number of new cases/total pop	# of new cases	Total pop	
Notes:			
7. Other information			
	Description		
Key conclusions of study			
References to other relevant studies			
Correspondence required for further information			
Other comments			
Notes:			

## Abbreviations

EPI: Expanded Programme on Immunisation
HAV: hepatitis A virus
HIV: human immunodeficiency virus
IgG: immunoglobulin G
IgM: immunoglobulin M
MDGs: Millennium Development Goals
MeSH: medical subject heading
SDGs: Sustainable Development Goals
WHO: World Health Organisation
WHOLIS: World Health Organisation Library Information System

## Footnotes

1 The water decade beginning from 2005 to 2015 and the sustainable sanitation: the drive to 2015 for sanitation launched in 2010 initiative
